# Extrapolating Weak Selection in Evolutionary Games

**DOI:** 10.1371/journal.pcbi.1003381

**Published:** 2013-12-05

**Authors:** Bin Wu, Julián García, Christoph Hauert, Arne Traulsen

**Affiliations:** 1 Evolutionary Theory Group, Max-Planck-Institute for Evolutionary Biology, Plön, Germany; 2 Department of Mathematics, University of British Columbia, Vancouver, British Columbia, Canada; University of Texas at Austin, United States of America

## Abstract

In evolutionary games, reproductive success is determined by payoffs. Weak selection means that even large differences in game outcomes translate into small fitness differences. Many results have been derived using weak selection approximations, in which perturbation analysis facilitates the derivation of analytical results. Here, we ask whether results derived under weak selection are also qualitatively valid for intermediate and strong selection. By “qualitatively valid” we mean that the ranking of strategies induced by an evolutionary process does not change when the intensity of selection increases. For two-strategy games, we show that the ranking obtained under weak selection cannot be carried over to higher selection intensity if the number of players exceeds two. For games with three (or more) strategies, previous examples for multiplayer games have shown that the ranking of strategies can change with the intensity of selection. In particular, rank changes imply that the most abundant strategy at one intensity of selection can become the least abundant for another. We show that this applies already to pairwise interactions for a broad class of evolutionary processes. Even when both weak and strong selection limits lead to consistent predictions, rank changes can occur for intermediate intensities of selection. To analyze how common such games are, we show numerically that for randomly drawn two-player games with three or more strategies, rank changes frequently occur and their likelihood increases rapidly with the number of strategies 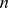. In particular, rank changes are almost certain for 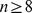, which jeopardizes the predictive power of results derived for weak selection.

## Author Summary

In evolutionary game dynamics in finite populations, selection intensity plays a key role in determining the impact of the game on reproductive success. Weak selection is often employed to obtain analytical results in evolutionary game theory. We investigate the validity of weak selection predictions for stronger intensities of selection. We prove that in general qualitative results obtained under weak selection fail to extend even to moderate selection strengths for games with either more than two strategies or more than two players. In particular, we find that even in pairwise interactions qualitative changes with changing selection intensity arise almost certainly in the case of a large number of strategies.

## Introduction

In evolutionary theory, weak selection means that differences in reproductive success are small. If fitness differences are close enough to zero, perturbation analysis allows to derive analytical results in models of population dynamics. This approach has a long standing history in population genetics, where selection is typically frequency independent [1]–[4]. More recently, weak selection has been introduced into evolutionary game theory [5]. If selection is weak, the outcomes of the game have only a small impact on fitness. Possible interpretations of this assumption include that the effects of the game under consideration are small or that it represents only one of many factors influencing reproductive success. A number of important analytical results have been derived using weak selection as well as rare mutations in finite populations [6]–[8].

In infinitely large populations, the intensity of selection merely results in a rescaling of time, but does not affect the outcome of the evolutionary dynamics [9], [10]. This means that long-term results under weak selection equally hold for arbitrary intensities of selection, provided the population is infinitely large. For finite populations it has been suggested that results obtained under weak selection may remain valid when the selection intensity is no longer weak [6], [8]. Here, we show that in general this is not the case. If population size is finite, the intensity of selection plays a decisive role and can qualitatively change the outcome.

**Figure 1:**
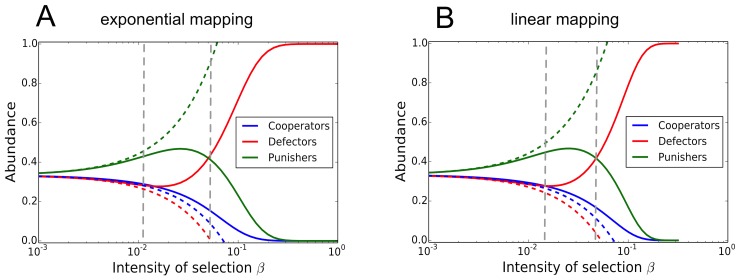
Average strategy abundance in compulsory public goods games with punishment [11], where 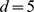, 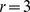, 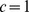, 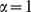 and 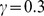. The game has three strategies: cooperators contribute to the common pool, defectors exploit cooperators, and altruistic punishers contribute to the common pool and punish defectors. The evolutionary dynamics are based on the Moran process in a population of size 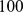, where an individual is chosen for reproduction with a probability proportional to its fitness 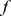, which is an increasing function of its payoff 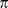. Weak selection implies that large payoff differences result in small fitness differences: **A** exponential payoff-to-fitness mapping, 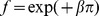 [20], [50] and **B** linear payoff-to-fitness mapping, 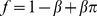 [11]. The dashed lines represents weak selection approximations. Vertical lines indicate the two selection intensities where the ranking of strategies changes. In both cases, most favored strategy changes at moderate intensities of selection. Thus, predictions based on weak selection results do not carry over to higher intensities of selection.

Let us illustrate this idea with an example. Consider the public goods game discussed in [11]. Therein, 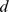 individuals are chosen from a population of size 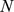 to play a public goods game. Individuals choose whether to contribute a fixed amount to a common pool at a cost 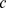. The amount in the common pool is multiplied by a positive factor 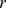 (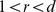) and distributed amongst all participants. The game considers three strategies: Cooperators, who contribute a fixed amount 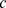 to a common pool, defectors, who do not contribute but benefit from the contributions of others, and punishers, who contribute 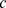 and pay a cost 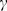 to impose a fine 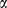 upon defectors. The game is devised to inspect the emergence of altruistic punishment, a behavior commonly found in human subjects [12]. The model assumes a standard Moran process [5], in which one individual is chosen proportional to fitness 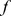 to reproduce and its offspring replaces a randomly chosen individual. Fitness is an increasing function of the payoff 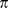 from the game, 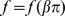, where 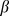 is the intensity of selection [13]. In addition, there is a small rate of mutations, such that a new mutant either goes extinct or reaches fixation before the next one occurs [14], [15]. This allows to approximate the dynamics by an embedded Markov chain on the monomorphic states, with fixation probabilities describing the transitions between those monomorphic states. The stationary distribution of this Markov chain allows to infer the relative abundance of different strategies. This approach is used frequently to describe evolutionary games in finite populations with more than two strategies [11], [16]–[19]. Figure 1 shows the strategy abundance in an imitation process for this public goods game with punishment. Panel 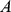 illustrates the outcome when payoffs are mapped onto fitness with an exponential function, 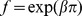, where 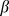 is the intensity of selection [20]. For weak selection altruistic punishment is the strategy most favored by selection, but this is not true for stronger selection. Moderate intensities of selection change the picture in favor of defection. This also holds when payoffs are mapped into fitness with the linear function 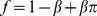 [5], as shown in Panel 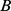. Changes in the ranking of strategies also occur for larger strategy sets [11], [17]–[19] but for a concise illustration of our point three strategies are sufficient.

In the example above, focusing only on the weak selection leads to results that do not even qualitatively hold for higher intensities of selection. The change in the order of strategies shows that, in this case, the predictive power of weak selection to higher intensities of selection is limited. However, many results on the selection of strategies are based on weak selection [8], [21]. In particular, in the context of the evolution of cooperation, simple analytical results derived under weak selection are popular [6], [7], [22]–[25]. However, based on the above example a number of questions arise: Are changes in the ranking of the frequencies of strategies a common occurrence as selection increases? What facilitates the change of ranks? The number of players? Or the number of strategies? Or does it depend on specific assumptions on the evolutionary dynamics? To answer these questions, we formally study imitation processes in symmetric games. Our results show that for games with two strategies, the ranking in strategy abundance can change with the intensity of selection, provided the number of players is more than two. Moreover, rank changes also arise in pairwise games with more than two strategies, and it is even highly likely in games with many strategies.

## Results

We study imitation dynamics in finite populations using pure strategies. While we could work with the Moran process discussed above, we choose for convenience a slightly different process, which is based on the pairwise comparison of two individuals. In this case, only payoff differences matter. Thus the effective parameter number is smaller (in a 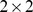 game, only two parameters are necessary and not four). This facilitates the analysis (see Lemma 3 in SI). Even for a Moran process with any payoff-to-fitness mapping [13], it is possible to establish payoff matrices that lead to rank changes. In this case, the same outline of proof applies, but involves a complicated multivariable analysis. For a pairwise comparison rule, a randomly chosen individual 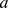 reassesses its strategy by a probabilistic comparison of its payoff 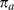 to that of a randomly selected model member 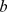 with payoff 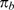. With a probability given by the imitation function 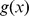 the focal individual adopts the model's strategy based on the payoff difference between the focal individual and the model, 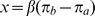, where 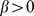 is the selection intensity. We assume that 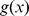 is a well defined probability for all real values of 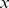. We further use the popular assumption that mutations are rare [11], [17], [26]–[29], such that populations are almost always monomorphous [14], [15]. We follow the traditional convention that mutants are restricted to a known finite set of strategies (similar to the finite allele model in population genetics). The stochastic dynamics is approximated by an embedded Markov chain with as many states as strategies in the game (see Methods). The stationary distribution of the associated Markov chain is a function of the intensity of selection, 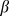, and yields a ranking of strategies such that the most abundant strategy is ranked first, the second most abundant strategy is ranked second, and so on. In the limit of vanishing selection, 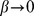, the payoff from the game does not matter and all strategies have equal abundances. Increasing intensity of selection makes some strategies more successful than others. This reflects appropriately in how strategies are ranked by the stationary distribution. We assume that in the strong selection limit, 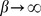, even the strategy of only slightly better performing model individuals is adopted with certainty, 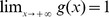 and that, similarly, the strategy of only slightly worse performing model indviduals is never adopted, 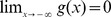.

### Games with two strategies

The abundance ranking of strategies is invariant under changes of the selection intensity in 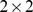 games for any imitation process [30]. However, for the Moran process with arbitrary payoff-to-fitness mappings, this does not necessarily hold: For example, in a Moran process with linear payoff-to-fitness mapping, such effects can appear in games with negative payoff entries when the intensity of selection approaches its maximal value, as the transition probabilities can approach zero in this case, leading to rapid changes of the fixation probability.

**Figure 2:**
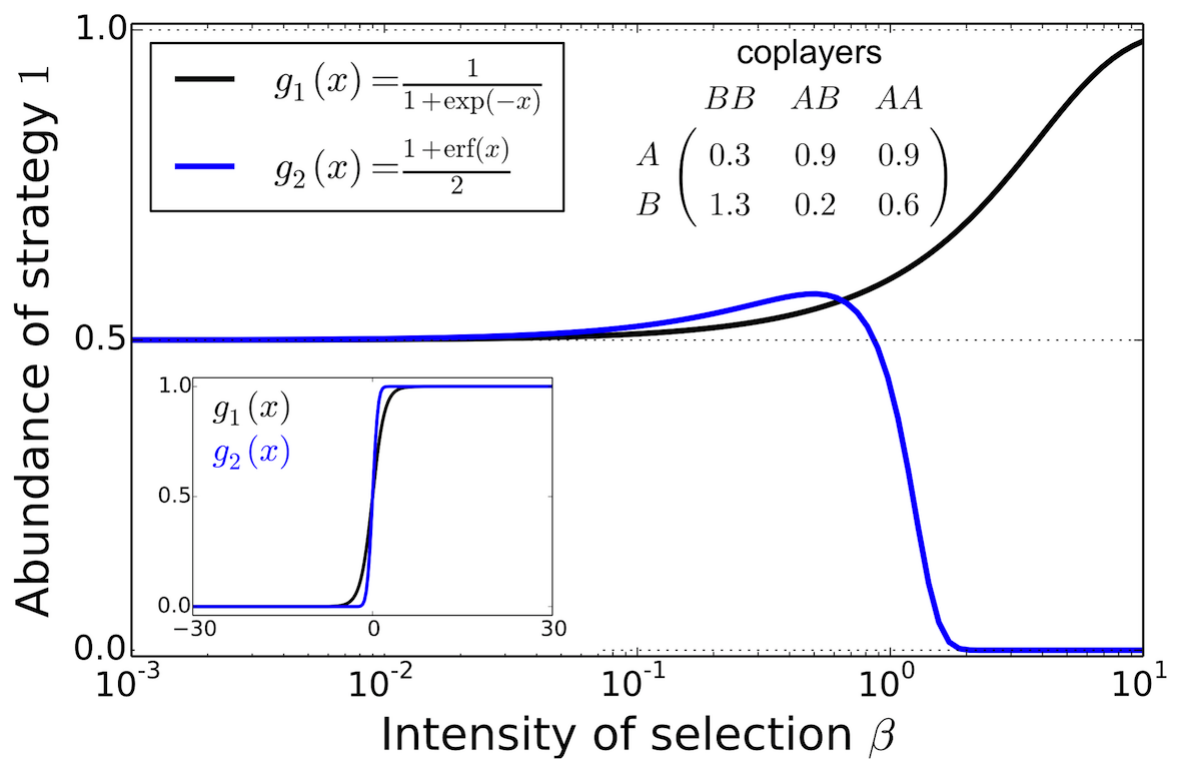
The rank invariance property is sensitive to the imitation function for two-strategy multiplayer games. We depict the average abundance of strategy 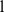 in a 2-strategy 3-player game in a population of size 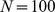 as a function of selection intensity 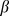 for two imitation functions, 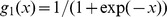 and 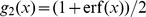, where 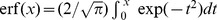 is the error function (see inset). The game is given by the table in the figure. Invariance of ranking holds if and only if the curves never cross the 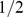 threshold. This threshold is crossed for imitation function 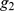 but not for 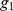, despite their similarity, see main text for details.

Figure 2 shows that rank changes can readily arise for simple imitation processes in games with three players, i.e., the minimal group size of multiplayer games. In this example, the ranking derived under weak selection carries over to any selection intensity for the Fermi imitation function, 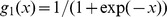. But for the rescaled error function 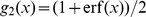, which represents a qualitatively similar imitation function, the ranking changes. It turns out that for any two-strategy game, the ranking invariance holds for the imitation function 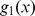, as a result of the special property 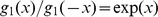. For the imitation function 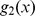, however, the criterion to determine that strategy 1 is more abundant under weak selection differs from that under strong selection. Section 3 of the SI shows technical details of these results.

Why do similar functions lead to radically different results when selection is not weak? The intuition behind is as follows: As shown in the SI, the stationary distribution depends only on the product 
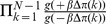
. Here 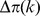 is the payoff difference between strategy 1 and strategy 2, where 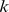 is the number of strategy 1 individuals in the population. The ranking can change when both the product in the enumerator and the product in the denominator converge to zero with increasing intensity of selection 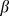. In this case, not the imitation function, but its first derivative or potentially its higher derivates far from zero matter, based on L'Hopital's rule.

In the SI, we show that even the monotonicity in the payoff difference cannot ensure the invariance of ranking for any two-strategy game and any imitation function (see Section 3 in SI). Yet this monotonicity applies for all 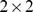 games, where the invariance property holds for any imitation function [30]. Therefore, we conclude that in general, ranking invariance does not hold for two-strategy games with arbitrary imitation processes. Since such multiplayer games have only become popular recently [31]–[36], this result may not be particularly surprising. However, in the next Section we show that even for 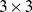 games between two players, ranking changes can occur.

### Games with three strategies

For games with more than two strategies, i.e., 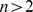, the problem is harder to tackle, because the stationary distribution does no longer depend on a single ratio of fixation probabilities, but becomes a more intricate rational function of all 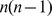 fixation probabilities, see e.g. [11]. At first, we restrict ourselves to 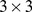 games and show that weak selection results do not carry over to stronger selection. Numerically we establish that this phenomenon occurs very often in the case of games with randomly drawn payoff matrices.

An example in which the ranking of strategies changes with the intensity of selection was already provided in the introduction. To go one step further, we provide a theorem for a more challenging constraint in which the limits of both weak and strong selection are identical, yet rank changes occur at intermediate selection strengths.

**Theorem 1** *Consider any imitation process with a strictly increasing, twice differentiable imitation function* 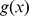 *. For a sufficiently large population size* 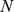 *and any selection intensity* 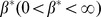 *, there exists a* 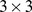 *payoff matrix* 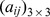 *with the following two properties:*

1. *The stationary distribution is uniform for* 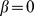 *(as always) and for* 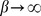 *.*
2. *At* 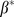 *, at least two strategies change their ranking.*

Theorem 1 states that weak selection results cannot be extrapolated to non-weak selection for 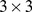 games (for a proof by construction see Section 4 of the SI). This implies that the ranking of strategies under weak selection has limited predictive value for higher intensity of selection. The theorem also shows that even if both weak selection and strong selection limits lead to the same evolutionary outcome, the ranking of strategies can still change at an intermediate selection intensity. This precludes the robustness of conclusions based on both the weak selection approximation and the strong selection.

**Figure 3:**
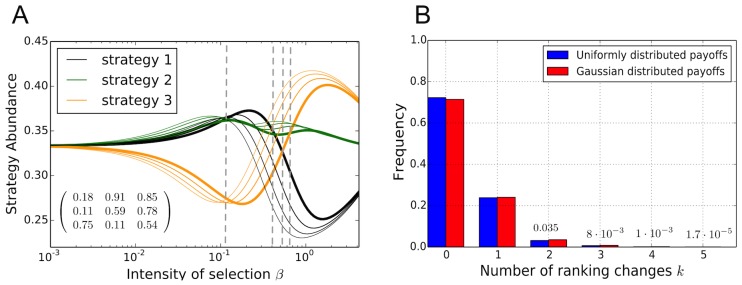
Number of changes in the abundance ranking of strategies in 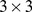 games. **A** Illustration of a particular game where selection curves intersect 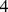 times, giving rise to 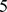 different rankings (from right to left population sizes 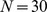 – thick lines, 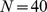, 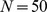, 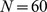
–thinner lines). **B** Statistics over the number of rank changes in games with randomly drawn payoff entries. At least one rank change is obtained in about one quarter of random games. The frequency of 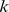 rank changes decreases approximately exponentially with 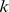. As an imitation function, we used the Fermi function 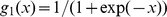. Parameters: Uniform distribution with payoff values in (0,1), Gaussian distribution with mean 0 and variance 1, frequencies obtained by averaging over 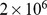 independent samples.

In order to determine how frequent such rank changes occur or how generic these games are, we analyze changes in the ranking of strategies in random games [36]–[39]. In particular, we compute the probability that rank changes occur and determine the number of changes in the rank of strategies, see Figure 3. The numerical procedure generates a random 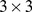 matrix, where each entry is drawn independently from a Gaussian distribution with zero mean and variance one or a uniform distribution over the interval 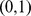. Strictly speaking, our numerical results are restricted to these two sampling distributions for the payoffs. However, the results suggest that the distribution has only a small influence on the number of rank changes as shown in Figure 3B. We compute the strategy abundances for an imitation process in the interval for 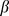 in 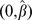, where 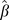 is chosen maximally while preventing numerical overflows. We then count the number of rank changes between all pairs of strategies. Note that the proof of Theorem 1 shows implicitly that in random games a simultaneous rank change of all three strategies occurs with probability measure zero. This is because these games are located on a subspace with a lower dimension than the space of games with intersections of pairs of strategies (see Section 4.1 in SI).

Figure 3A shows an example where a randomly generated game results in four rank changes. This illustrates that the ranking obtained for weak selection cannot be used to extrapolate to non-weak selection. The commonness of rank changes is estimated based Monte Carlo simulations, see Figure 3B. With a probability greater than 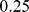 at least one rank change occurs but the likelihood decreases rapidly with the number of rank changes.

The numerical approach shows that the construction provided in Theorem 1 is relevant for a substantial fraction of random games and does not merely represent a non-generic, special case. It also shows that a larger number of rank changes may occur as illustrated in Figure 3.

### Games with more than three strategies

Theorem 1 states that 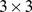 games exist in which the strategies change their ranking in abundance. Naturally this also holds for games with more strategies. To determine the probability and numbers of such rank changes in random 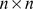 games [36]–[39], we generalize the numerical procedure described above.

**Figure 4:**
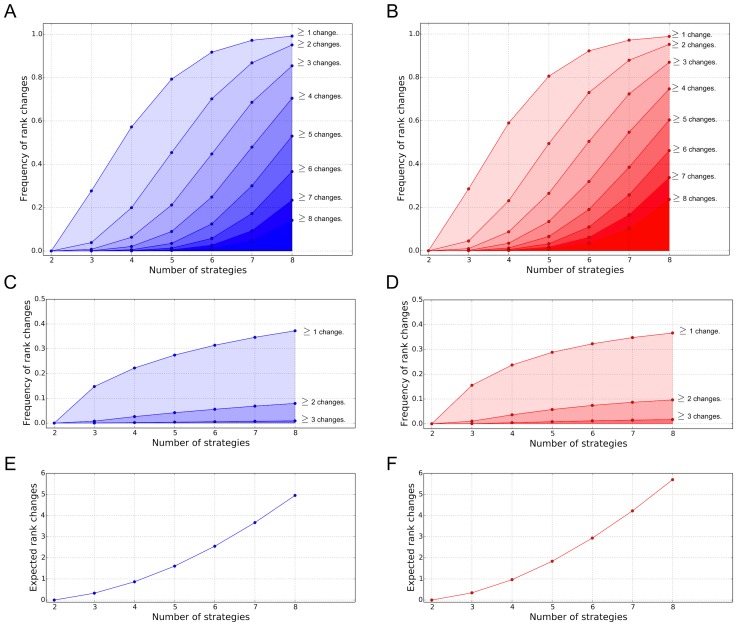
Occurrence of rank changes in random games. In the first row, we plot the estimated probability 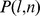 of getting at least 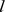 rank changes as a function of the number of strategies 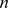 for uniformly distributed payoffs (Panel A) and Gaussian distributed payoffs (Panel B). In the second row, we plot the estimated probability of getting at least 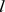 changes in the most abundant strategy as a function of the number of strategies 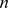 for uniformly distributed payoffs (Panel C) and Gaussian distributed payoffs (Panel D). Finally, on the third row we show the expected total number of rank changes for uniformly and Gaussian distributed payoffs (Panels E and F). Here, we used a Fermi imitation function 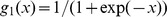 in a population of size 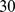. Simulations: For each 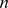, (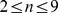), 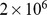 random 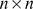 matrices are sampled.

Games with more than 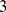 strategies increase in complexity and, as expected, increasing 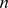 leads to more rank changes. Let 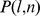 be the probability that at least 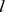 changes in the abundance ranking occur in random 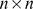 games. Figure 4 shows that 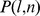 increases rapidly with the number of strategies 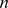 if we assume that the entries of the payoff matrix are sampled from either a uniform or a Gaussian distribution. For 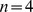, the probability that the ranking derived under weak selection is not valid for higher selection intensity already exceeds one half. For 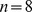, it is almost 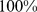. The numerical investigation of random games shows that with many available strategies, the stationary distribution computed for weak selection can be very different from the stationary distribution obtained for larger intensities of selection. This is of particular relevance in applications where behavioral diversity is important [18], [26].

Similarly, the expected number of rank changes for random 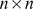 games also increases with 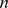, see Figure 4. In particular, for 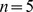, the expected number of rank changes is already more than one. Hence, for games with many strategies it is very likely that the stationary distribution obtained under weak selection is qualitatively quite different from the stationary distribution obtained for stronger selection.

## Discussion

For two-strategy multiplayer games in well-mixed populations under small mutation rates [14], [15], we have shown that the ranking of the average strategy abundance derived for weak selection may change when increasing selection strength. Moreover, the ranking is sensitive to the details of the evolutionary process, such as the choice of imitation functions.

In evolutionary games in finite populations the assumption that mutation rates are sufficiently rare to consider pairwise invasions between strategies is popular [11], [17], [26]–[29], [40] and often the only analytically feasible approach. However, it remains challenging to interpret the analytical results for the stationary distribution for all selection intensities [11]. Therefore, weak selection approximations [5], [22] or strong selection limits [17], [19], [27] are often used to obtain simpler analytical results that are easier to interpret.

Here, we have shown that already for 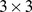 games, attempts to extrapolate results derived in one of those simplifying cases may often fail because even the qualitative features of the stationary distribution, i.e. the ranking of strategy abundances, may change as a function of the selection strength. In particular, the strategy with highest abundance may change with the intensity of selection. In fact, even considering the two limiting cases of the selection intensity together is not enough. Our results show that even if weak and strong selection limits lead to the same ranking, other rankings can still arise for intermediates selection intensities. Thus, we conclude that even though these extreme cases are insightful, abundances at intermediate selection intensity levels have to be considered as well to establish the generality of the results and robustness of the conclusions.

An intuitive reason for changes in the abundance ranking of strategies for 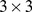 games in both the weak and strong selection is based on risk dominance. For strong selection, the pairwise probability current always flows towards the risk dominant strategy [14], whereas for weak selection, the average abundance is based on the sum of the risk dominance conditions between all different strategies [41].

We have focused solely on well-mixed populations and our analytical considerations cannot easily be generalized to structured populations. However, several papers on the evolution of cooperation have shown that the ranking of the average abundance of strategies can change in structured populations even in 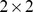 games [42]–[45]. Thus, this issue is also of interest in structured populations, where the weak selection approximation is particularly powerful [21], [24], [25], [46], but for example fails to predict the potential decrease of cooperation in the spatial snowdrift game [42].

Our results have been obtained for imitation processes, i.e. processes in which one individual probabilistically compares its performance to another one and tends to adopt strategies of better performing members of the population. The results derived for three or more strategies assume rare mutations such that the transition matrix of the embedded Markov chain 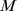 only depends on the fixation probabilities of pairs of strategies. Therefore, all our results immediately carry over to the Moran process with exponential payoff-to-fitness mapping [20], [47], because such a Moran process has the same fixation probability as the imitation process with imitation function 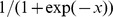 for any intensity of selection [20]. This fact illustrates that the existence of such rank changes do not depend on the details of the microscopic evolutionary process, but are a generic feature of evolutionary games in finite populations.

## Methods

We assume a finite well-mixed population of size 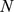. For two player games, individuals interact in pairs according to a symmetric game given by the 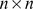 matrix 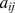 where 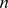 denotes the number of strategies. A player with strategy 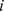 playing against strategy 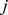 gets payoff 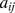. Payoffs are computed for every individual assuming everyone interacts with everyone else in the population. For the multiplayer case, we follow the notation from [36]. Selection acts by comparing the payoffs of two randomly chosen individuals. Individual 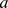 with payoff 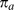 adopts the strategy of individual 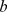 with payoff 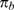 with probability 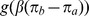, where 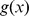 is called the imitation function. In an evolutionary process individuals must be more likely to imitate a strategy that performs better and hence we assume that 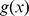 is increasing, 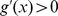 for all 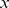, and for technical reasons we require that 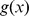 is continuously differentiable (with the exception of the proof of Theorem 1, which requires twice continuous differentiability). This implies that strategies achieving higher payoffs have a higher probability of being represented in the next generation. The intensity of selection is 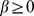. If 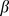 approaches zero, payoff differences have vanishingly small effects on selection. We also assume that 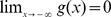 and 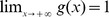, which means that for infinite intensity of selection, only the sign of 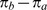 matters.

Variation in the population is generated by mutations. That is, the imitation step described above happens with probability 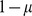. With probability 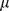, a mutation occurs and the focal individual adopts a uniformly chosen strategy. Without mutations it is possible to compute the fixation probability 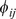, of a mutant playing strategy 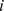 in a population of 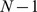 individuals playing 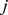 [5]. For small 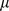, the dynamics is approximated by an embedded Markov chain [14], [15] with an 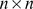 transition matrix 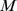 that is fully determined by the 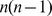 different fixation probabilities 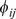 (Section 2 SI).

In a large class of evolutionary processes (where all transitions between states are possible), the transition matrix 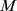 has a unique stationary distribution for every 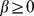. More precisely, the stationary distribution is unique whenever the Markov chain is irreducible and aperiodic, and characterizes the average abundance of each strategy in the long run [48], [49]. The evolutionary outcome we are interested in is the ranking based on the average abundance on the set of strategies 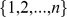. The stationary distribution of 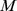 is the the uniform distribution 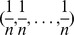 for 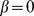. For weak selection, we obtain the ranking over the 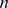 strategies by ordering the derivatives of the components of the stationary distribution at 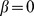.

For the computational results, we determine the strategy abundances for a random game as a function of the selection strength, 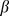, over the interval 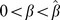, where 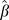 is dynamically adjusted to avoid arithmetic overflow. We then count the number of rank changes between any pair of strategies, i.e. changes in their relative abundance for 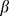 in 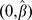. Random games are constructed by sampling payoffs from an independent identical distribution, which is either uniform or Gaussian. Averages are taken over 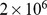 samples in all cases.

Our source code in Python is publicly available on figshare (http://dx.doi.org/10.6084/m9.figshare.814470).

## Supporting Information

Text S1 Supplementary Information: Extrapolating weak selection in evolutionary games.
(PDF)
