# Supplementary material for: Extrapolating Weak Selection in Evolutionary Games
Source: PLoS Comput Biol. 2013 Dec 5;9(12):e1003381. doi: 10.1371/journal.pcbi.1003381 (PMC3854678; doi:10.1371/journal.pcbi.1003381)
Supplement: Text S1 — Supplementary Information: Extrapolating weak selection in evolutionary games. (PDF) [file pcbi.1003381.s001.pdf]

Supplementary Information: Extrapolating weak selection in evolutionary games

Bin Wu[1*], Julián García[1], Christoph Hauert[2], Arne Traulsen[1]
1 Evolutionary Theory Group, Max-Planck-Institute for Evolutionary Biology, Plön, Germany
2 Department of Mathematics, University of British Columbia, Vancouver, British Columbia, Canada
∗ **E-mail: Corresponding bin.wu@evolbio.mpg.de**

Contents

1 Preliminaries **2**

2 Embedded Markov chain approximation for weak mutation **2**

3 Discussion of the ranking invariance property for two-strategy multiplayer games **3**

4 Proof of Theorem 1 **4**
4.1 Construction of the 3×3 matrix . . . 4
4.2 Proof that the established matrix satisfies the constraints . . . 5

5 Proofs of Lemmas **7**
5.1 Lemma 1 . . . 7
5.2 Lemma 2 . . . 9
5.3 Lemma 3 . . . 14
5.4 Lemma 4 . . . 15
5.5 Lemma 5 . . . 16
5.6 Lemma 6 . . . 16

1 Preliminaries

We use the following symbols:

N	population size
n	number of strategies in the game
a_{ij}	payoff of strategy i player when facing a strategy j player
β	selection intensity
$\Delta\pi(k)$	payoff difference between mutant and wild types when there are k mutants
$g(x)$	imitation function, where $x = \beta\Delta\pi(k)$
ϕ_{ij}	fixation probability of a single mutant of type j taking over a wild population of type i

In well-mixed populations, every individual plays equally likely with all the other players in the populations. In 2×2 games with payoff matrix $(a_{ij})_{2\times 2}$, the payoffs for strategy 1 and 2 are thus given by $\pi_1(k) = (a_{11}(k-1) + a_{12}(N-k))/(N-1)$ and $\pi_2(k) = (a_{21}k + a_{22}(N-k-1))/(N-1)$, where k is the number of strategy 1 individuals. The payoff difference $\Delta\pi(k) = \pi_1(k) - \pi_2(k)$ is given by

$$\underbrace{\frac{a_{11} - a_{12} - a_{21} + a_{22}}{N-1}}_{u_{12}} k + \underbrace{\frac{-a_{11} + Na_{12} - a_{22}N + a_{22}}{N-1}}_{v_{12}} \tag{1}$$

Note that the fixation probability ϕ_{ij} depends only on $g(\beta\Delta\pi)$. In turn, $\Delta\pi$ depends on u_{ij} and v_{ij}. Moreover, since $u_{ji} = u_{ij}$ and $v_{ji} = -Nu_{ij} - v_{ij}$, $G_{ij} = \phi_{ij} - \phi_{ji}$ only depends on u_{ij}, v_{ij} and β, and can be written as a function $G_{ij}(\beta, u_{ij}, v_{ij})$.

For convenience, when dealing with games of two strategies, we drop the indexes in u_{ij}, v_{ij}, G_{ij}. In addition, the indexes are treated modulo the number of strategies n if we are considering more than two strategies.

2 Embedded Markov chain approximation for weak mutation

Under sufficiently weak mutation a mutant fixates or goes extinct before a new mutant arises [1,2]. This means that the population spends most of the time in monomorphic states where all the individuals are of the same strategy. The dynamics in this case is approximated by the transition probabilities ϕ_{ij} between homogeneous states i and j. Thus, the dynamics is fully approximated by an embedded Markov chain with transition matrix M, given by

$$\begin{array}{c} \\ \text{All } 1 \\ \text{All } 2 \\ \vdots \\ \text{All } n \end{array}
\begin{array}{c} \begin{array}{cccc} \text{All } 1 & \text{All } 2 & \dots & \text{All } n \end{array} \\
\begin{pmatrix} 1 - \frac{\mu}{n}\sum_{j\neq 1}\phi_{1j} & \frac{\mu}{n}\phi_{12} & \dots & \frac{\mu}{n}\phi_{1n} \\ \frac{\mu}{n}\phi_{21} & 1 - \frac{\mu}{n}\sum_{j\neq 2}\phi_{2j} & \dots & \frac{\mu}{n}\phi_{2n} \\ \vdots & \vdots & \vdots & \vdots \\ \frac{\mu}{n}\phi_{n1} & \frac{\mu}{n}\phi_{n2} & \dots & 1 - \frac{\mu}{n}\sum_{j\neq n}\phi_{nj} \end{pmatrix} \end{array}, \tag{2}$$

where All i denotes the state consisting of only strategy i individuals, and ϕ_{ij} is the fixation probability of a single mutant of type j in a population of $N-1$ i individuals. Note that we will always follow the common assumption of uniform mutation kernels [3].

To compute the fixation probabilities ϕ_{ij} we use a generalized pairwise comparison rule. Each individual interacts with the rest of the population equally likely and obtains a payoff. Then one individual is selected randomly to imitate another randomly chosen individual's strategy with a probability $g(\beta\Delta\pi)$, where $\Delta\pi$ is the payoff difference between its opponent and the focal individual.

Let k denote the number of the mutants in the population. The transition probabilities to go from state k to $k \pm 1$, $T_k^{\pm}$ are given by

$$\begin{aligned} T_k^+ &= \frac{N-k}{N}\frac{k}{N} g(+\beta\Delta\pi(k)), \\ T_k^- &= \frac{N-k}{N}\frac{k}{N} g(-\beta\Delta\pi(k)), \end{aligned} \tag{3}$$

while the probability to stay in state k is $1 - T_k^+ - T_k^-$. The fixation probability is given by

$$\phi_{ij} = \frac{1}{1 + \sum_{m=1}^{N-1} \prod_{k=1}^{m} \frac{T_k^-}{T_k^+}}, \tag{4}$$

where i and j refer to the wild and mutant types [4–6].

Note that the fixation probabilities in M depend on the intensity of selection β. We compute the stationary distribution of M, given by the left eigenvector of M to the unit eigenvalue. This stationary distribution is also a function of selection intensity, a vector with n elements where each element is the long-term abundance of the corresponding strategy. We are interested in how the ranking of strategies according to abundance changes with increasing selection intensity.

3 Discussion of the ranking invariance property for two-strategy multiplayer games

For 2×2 games, it has been shown that the ranking of strategies is invariant with increasing selection intensity [7]. This is valid for any pairwise comparison rule. No technical constraints in the imitation function $g(x)$ are required, thus the result is robust if the payoffs are given by two-strategy two-player games.

Let strategy 1 and 2 be the mutant and wild type respectively. For rare mutations, the transition matrix M in Eq. (2) is

$$\begin{pmatrix} 1 - \frac{\mu}{2}\phi_{12} & \frac{\mu}{2}\phi_{12} \\ \frac{\mu}{2}\phi_{21} & 1 - \frac{\mu}{2}\phi_{21} \end{pmatrix}, \tag{5}$$

and the stationary distribution is $(\frac{\phi_{21}}{\phi_{12}+\phi_{21}}, \frac{\phi_{12}}{\phi_{12}+\phi_{21}})$ [2,8]. The ratio between the abundance of strategy 1 and 2 is ϕ_{21}/ϕ_{12}. Following [8–10] we have

$$\begin{aligned} \frac{\phi_{21}}{\phi_{12}} &= \prod_{k=1}^{N-1} \frac{T_k^+}{T_k^-} \\ &= \prod_{k=1}^{N-1} \frac{g(+\beta\Delta\pi(k))}{g(-\beta\Delta\pi(k))} \\ &= \exp\left[\underbrace{\sum_{k=1}^{N-1} \ln\left(g(+\beta\Delta\pi(k))\right) - \ln\left(g(-\beta\Delta\pi(k))\right)}_{D_1}\right], \end{aligned} \tag{6}$$

where, in the last step, we have used $\prod \exp x = \exp \sum x$. By Eq. (6), for $D_1 > 0$, strategy 1 is more abundant while strategy 2 is more abundant for $D_1 < 0$. For $D_1 = 0$, the two strategies are equally

abundant. Thus the invariance of ranking with increasing selection intensity is equivalent to the invariance of sign for D_1 for all $\beta > 0$. For D_1, we have the following properties: (i) For the Fermi imitation function, $g(x) = 1/(1+\exp(-x))$, $D_1 = \beta \sum_{k=1}^{N-1} \Delta\pi(k)$, this leads to the invariance of signs for all the $\beta > 0$ for any two-strategy games. Thus for any two-strategy game, the ranking invariance property holds for the Fermi process; (ii) D_1 is invariant by rearranging the index k, thus the ranking is invariant by rearranging k such that the rearranged $\Delta\pi(k)$ is monotonic; (iii) For weak selection, D_1 is approximated by $\beta(2g'(0)/g(0)) \sum_{k=1}^{N-1} \Delta\pi(k)$. Since $g'(0)/g(0) > 0$ for all imitation functions, the sign of D_1 is solely determined by the sum of the payoff differences. This illustrates that under weak selection, for any given two-strategy game, the ranking is the same for any imitation process.

In contrast, for games with more than two players, the abundance ranking may change as illustrated in the main text for a three player game. In addition to the Fermi function, we discuss the imitation function given by $g(x) = (1 + \text{erf}(x))/2$, where $\text{erf}(x) = (2/\sqrt{\pi}) \int_0^x \exp(-t^2)dt$ is the error function. In this case, we find that the ranking can change with the selection intensity. It turns out that the criterion to determine the ranking differs between weak and strong selection for this imitation function. For strong selection, expanding $g(1/t)$ around $t = 0^+$ leads to the approximation of $g(x)$ for $x \to +\infty$, i.e. $g(x) \approx 1 - (2/\sqrt{\pi})x\exp(-x^2)$. Similarly, we have $g(x) \approx -(2/\sqrt{\pi})x\exp(-x^2)$ for $x \to -\infty$. Thus under strong selection, or sufficiently large $|x|$, $\ln(g(x)) - \ln(g(-x)) = \ln(g(x)/g(-x)) \approx \text{sgn}(x)\ln(\frac{\sqrt{\pi}}{2}\frac{\exp(x^2)}{|x|} - 1)$. Considering that $\frac{\exp(x^2)}{|x|} \to +\infty$ as $|x| \to +\infty$, $\ln(g(x)) - \ln(g(-x)) \approx \text{sgn}(x)\left(x^2 - \ln(|x|) + \ln(\frac{\sqrt{\pi}}{2})\right)$ as $x \to +\infty$. Since the leading term is $\text{sgn}(x)x^2$, for strong selection, the sign of D_1 is determined by $\sum_{k=1}^{N-1} \text{sgn}(\Delta\pi(k))(\Delta\pi(k))^2$. For weak selection, however, the sign of D_1 is determined by $\sum_{k=1}^{N-1} \Delta\pi(k)$ as aforementioned. In fact, the multiplayer game is constructed such that the sign differs between $\sum_{k=1}^{N-1} \Delta\pi(k)$ and $\sum_{k=1}^{N-1} \text{sgn}(\Delta\pi(k))(\Delta\pi(k))^2$.

This example shows that for multiplayer games the ranking invariance property does not always hold as it does for pairwise games. Furthermore, D_1 is invariant by rearranging the index k in Eq. (6) such that the rearranged $\Delta\pi(k)$ is monotonic as in 2×2 games, hence even monotonicity in payoff differences is not sufficient to ensure the invariance of rank for any pairwise comparison rules. This implies that the invariance of rank is not robust for two-strategy multiplayer games.

4 Proof of Theorem 1

Theorem 1 *Consider any imitation process with a strictly increasing, twice differentiable imitation function $g(x)$. For a sufficiently large population size N and any selection intensity $\beta^*(0 < \beta^* < \infty)$, there exists a 3×3 payoff matrix $(a_{ij})_{3\times 3}$ with the following two properties:*

1. *The stationary distribution is uniform for $\beta = 0$ (as always) and for $\beta \to \infty$.*
2. *At β^*, two strategies change their ranking.*

Theorem 1 implies that the rank can still change for moderate selection intensity, even when weak and strong selection limits lead to the same rank. Based on the first three lemmas that follow, we construct a 3×3 payoff matrix that satisfies the two conditions in Theorem 1.

4.1 Construction of the 3×3 matrix

The intuition to establish these lemmas is as follows: We need to find a 3×3 matrix (or nine payoff entries) to satisfy the constraints in Theorem 1. Lemma 3 formally establishes that only six parameters $u_{i,i+1}, v_{i,i+1}$, $i = 1, 2, 3$ are necessary for pairwise comparison processes. To further reduce the number of parameters, Lemma 1 is introduced. Therein, it establishes a mapping between $u_{i,i+1}$ and $v_{i,i+1}$, i.e., $v_{i,i+1} = v(u_{i,i+1})$, $i = 1, 2, 3$. In addition, Lemma 2 is introduced to clarify the domain where $v(u)$ is

defined. As a consequence, only three parameters $u_{i,i+1}$, $i = 1, 2, 3$ are required. This is smaller than the dimension of a 3×3 matrix. It suggests that the matrices satisfying the constraints in Theorem 1 are located on a subspace with the dimension lower than that of a 3×3 matrix.

Lemma 2 assumes that the imitation function is twice continuously differentiable and population size large. Consequently, Theorem 1 requires the same assumptions to use the inverse function theorem. Yet the example shown in Figure. 1 suggests that assumption of large population size is not necessary.

Lemma 1 *For every imitation process with strictly increasing continuously differentiable imitation function* $g(x)$ *with* $\lim_{\beta\to-\infty} g(x) = 0$ *and* $\lim_{\beta\to+\infty} g(x) = 1$, $\beta^* > 0$, *population size* N *and* $-1 < c^* < 1$, *there is a* $\delta > 0$ *and a continuously differentiable function* $v = v(u)$ *defined in* $(-\delta, \delta)$ *such that* $G(\beta^*, u, v(u)) = c^*$. *Furthermore, if* $0 < c^* < 1$, *then* $v(0) > 0$. *(The proof is given in Section 5.1.)*

Lemma 2 *For any imitation process with strictly increasing second order continuously differentiable imitation function* $g(x)$ *and any* $\beta > 0$, *if* $v_0 > 0$, *then for large population size* N, *there is a* $\delta > 0$ *such that* $H(u,v) = (\frac{\partial}{\partial\beta}G, G)^T$ *is invertible in a vicinity of* $(0, v_0)$ *with radius* δ, $B((0,v_0),\delta) = \{(u,v)|\sqrt{u^2 + (v - v_0)^2} < \delta\}$. *(The proof is given in Section 5.2).*

Lemma 3 *If there are* 3 *strategies in the population, for every* $u_{i,i+1}$, $v_{i,i+1}$, $i = 1, 2, 3$, *and population size* N, *there is an affine space of dimension* 3, *such that every element in such a space corresponds to a payoff matrix* $(a_{ij})_{3\times3}$. *(The proof can be found in Section 5.3).*

Steps to establish the matrix:

1. For the given $\beta^* > 0$, $g(x)$ and N, we arbitrarily choose $0 < c^* < 1$. By Lemma 1, there is a $\delta_1 > 0$ and an implicit function $v(u)$ defined in $u \in (-\delta_1, \delta_1)$ such that $G(\beta^*, u, v(u)) = c^*$. Since $c^* > 0$, again by Lemma 1, we have $v(0) > 0$. Considering that $v(u)$ is continuous at $u = 0$, there exists a $\delta_2 > 0$, such that $v(u) > 0$ for all $u \in (-\delta_2, \delta_2)$.

2. By Lemma 2, there is a δ_3 such that $H(u,v)$ is locally invertible in the vicinity of $(0, v(0))$ with radius δ_3. We denote $\delta = \min\{\delta_1, \delta_2, \delta_3\}$ and randomly take $u_{i,i+1} \in (0, \delta)$, $i = 1, 2, 3$, such that $0 < u_{1,2} < u_{2,3} < u_{3,1} < \delta$.

3. Based on $u_{i,i+1}$, $v_{i,i+1}$, $i = 1, 2, 3$, (six numbers in total), by Lemma 3, there exists an affine space, such that every element in such a space corresponds to a 3×3 matrix (a_{ij}).

Remark For the established game matrix, $u_{i,i+1}$ and $v_{i,i+1}$ $(i = 1, 2, 3)$ are all positive. In fact, the second step explicitly illustrates that $u_{i,i+1} > 0$ for $i = 1, 2, 3$. In addition, considering that $v(0) > 0$, by the continuity of $v(u)$ in the interval $(0, \delta)$, where $u_{i,i+1}$ lie, $v_{i,i+1} = v(u_{i,i+1}) > 0$. In addition, $G(\beta^*, u_{i,i+1}, v_{i,i+1}) = c^*$ for $i = 1, 2, 3$.

Section 4.2 proves that any 3×3 game that follows the recipe above fulfills the conditions in Theorem 1. An example using the Fermi function is shown in Figure. 1.

4.2 Proof that the established matrix satisfies the constraints

In order to prove that the matrix established in Section 4.1 fulfills the conditions listed in Theorem 1, we list three more lemmas. They are sufficient conditions under which the constraints in the Theorem are fulfilled: Lemma 4 and Lemma 6 illustrate conditions under which the ranking is uniform and the ranking changes respectively, reflecting the two main constraints in Theorem 1. While Lemma 5 is a sufficient condition under which the uniform distribution is the stationary distribution for strong selection limit. Then we prove that the constructed matrix satisfies the conditions in these lemmas, which completes the proof.

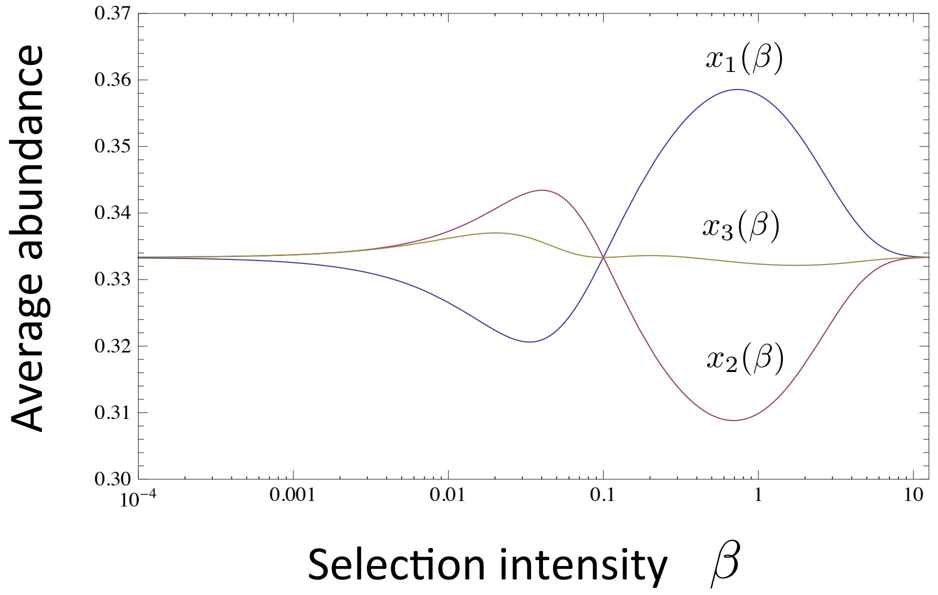

S1.pdf

Figure 1. We use a Fermi process with imitation function $g(x) = 1/(1 + \exp(-x))$. Let $N = 30$, $c^* = 0.1$, $\beta^* = 0.1$ and $u_{i,i+1} = i/(2N)$, where $i = 1, 2, 3$. Using the procedure above we find a game such that the uniform distribution is the stationary distribution for $\beta = 0$ and $\beta \to \infty$, and two of the three strategies exchange the rank at $\beta^* = 0.1$ as Theorem 1 states. Furthermore, we observe that (i) every two strategies exchange their rank at the $\beta^* = 0.1$; (ii) the most abundant strategy becomes the least abundant when the selection intensity exceeds this critical value β^*. (iii) $\beta^* = 0.1$ is the unique positive selection intensity at which all three strategies are equal in abundance.

Lemma 4 *For $n = 3$ and $\beta^* > 0$, the uniform distribution is the stationary distribution of the transition matrix M at β^* if and only if $G_{1,2} = G_{2,3} = G_{3,1}$ at β^*. (For a proof, see Section 5.4.)*

This lemma demonstrates that the uniform distribution is the stationary distribution if and only if the relative transition rates between every two strategies are identical.

Lemma 5 *For a 2×2 payoff matrix (a_{ij}) and any imitation process with strictly increasing function $g(x)$ with $\lim_{x\to-\infty} g(x) = 0$ and $\lim_{x\to+\infty} g(x) = 1$, if $u_{12} > 0$ and $v_{12} > 0$, then $\lim_{\beta\to+\infty} \phi_{12} = 1$ and $\lim_{\beta\to+\infty} \phi_{21} = 0$. (For a proof, see Section 5.5.)*

This lemma shows that for cyclic dominance of strategies, i.e., Rock-paper-scissors games, the stationary distribution converges to the uniform distribution in the strong selection limit.

Lemma 6 *For $n = 3$, assume that imitation function $g(x)$ is strictly increasing and continuously differentiable, if i) there exists a strategy $i \in \{1, 2, 3\}$ such that $\left(\frac{\partial}{\partial\beta} G_{i,i+1} - \frac{\partial}{\partial\beta} G_{i+1,i+2}\right)\Big|_{\beta^*} \neq 0$; ii) the uniform distribution is the stationary distribution at $\beta^* > 0$, then there are two strategies out of the three that exchange their ranking at β^*. (For a proof, see Section 5.6.)*

This lemma shows that if the uniform distribution is the stationary distribution at a certain selection intensity, a ranking change occurs, provided the increase rates of the relative transition probabilities at the selection intensity differ from each other.

We prove that the matrix established in Section 4.1 fulfills the conditions listed in Theorem 1.

Proof First, when the selection intensity is zero, $\phi_{i,i+1} = \phi_{i+1,i} = 1/N$, or $G_{i,i+1} = \phi_{i,i+1} - \phi_{i+1,i} = 0$ for $i =, 1, 2, 3$. By Lemma 4 the uniform distribution is the stationary distribution at $\beta = 0$.

Next we prove that for the matrix (a_{ij}), the uniform distribution is the stationary distribution when $\beta \to \infty$. By the Remark in Section 4.1, $u_{i,i+1}$ and $v_{i,i+1}$, $(i = 1, 2, 3)$ are all positive. Thus, by Lemma 5, $\phi_{i,i+1} \to 1$ and $\phi_{i+1,i} \to 0$ as $\beta \to \infty$. This yields that $G_{i,i+1} = \phi_{i,i+1} - \phi_{i+1,i} = 1$ for $i = 1, 2, 3$ as the selection intensity approaches $+\infty$. By Lemma 4, the uniform distribution is the stationary distribution when $\beta \to \infty$.

Finally, we prove that there are two strategies such that their rank alters at β^*. By the Remark in Section 4.1, $G(\beta^*, u_{i,i+1}, v_{i,i+1}) = c^*$ for $i = 1, 2, 3$, there exists strategy k^* such that $\frac{\partial}{\partial\beta} G(\beta^*, u_{k^*,k^*+1}, v_{k^*,k^*+1}) \neq \frac{\partial}{\partial\beta} G(\beta^*, u_{k^*+1,k^*+2}, v_{k^*+1,k^*+2})$. (Otherwise, there is l^* such that $\frac{\partial}{\partial\beta} G(\beta^*, u_{k,k+1}, v_{k,k+1}) = l^*$ for $k = 1, 2, 3$. Remembering $H = (\frac{\partial}{\partial\beta} G, G)^T$, we have $H(u_{i,i+1}, v_{i,i+1}) = (c^*, l^*)$ at β^*, for $i = 1, 2, 3$. Taking into account that $(u_{i,i+1}, v_{i,i+1})$ are chosen in the domain where $H(u, v)$ is invertible at β^* (Step 2 in the procedure to establish the matrix), we have $u_{1,2} = u_{2,3} = u_{3,1}$. This contradicts to the fact that $u_{1,2} < u_{2,3} < u_{3,1}$ (Step 2 in the above payoff establishment procedure). In addition, by the first procedure of the construction, we obtain that $G_{i,i+1}|_{\beta^*} = G(\beta^*, u_{i,i+1}, v(u_{i,i+1})) = c^*$ for $i = 1, 2, 3$. By Lemma 4, the uniform distribution is the stationary distribution of $\beta = \beta^*$. Therefore by Lemma 6, there are two strategies out of three whose rank exchanges at β^*.

This completes the proof.

Remark For the established matrix, the uniform distribution is also the stationary distribution when $\beta = \beta^*$. This is why Figure. 1 shows that all the three lines are intersecting at the given selection intensity β^*.

5 Proofs of Lemmas

5.1 Lemma 1

Proof The outline of the proofs are in the following: Given the imitation function $g(x)$, selection intensity $\beta^* > 0$, population size N and $-1 < c^* < 1$, for $u = 0$, there exits a unique v, denoted as v_0, such that

$G(\beta^*, 0, v_0) = c^*$. Then, based on implicit function theory, we prove the existence of the implicit function $v(u)$ defined in a vicinity of $u = 0$ such that $G(\beta^*, u, v(u)) = c^*$. For $c^* \geq 0$, we need to prove that (i) $G(\beta^*, u, v)$ is continuously differentiable as a function of u and v; (ii) $\frac{\partial G}{\partial v}$ is not zero at point $(0, v_0)$. For $c^* < 0$, we convert it into a case with $c^* > 0$.

First we prove that given the imitation function $g(x)$, selection intensity $\beta^* > 0$, population size N and $-1 < c^* < 1$, for $u = 0$, there exits a unique v_0 such that $G(\beta^*, 0, v_0) = c^*$.

The fixation probability of a single mutant of strategy j taking over the wild population of strategy i is given by [6]

$$\phi_{ij} = \frac{1}{1 + \sum_{i=1}^{N-1} \prod_{k=1}^{i} \frac{g(-\beta^*(uk+v))}{g(+\beta^*(uk+v))}}. \tag{7}$$

Considering that $\phi_{ji} = \phi_{ij} \prod_{k=1}^{N-1} \frac{g(-\beta^*(uk+v))}{g(+\beta^*(uk+v))}$, $G(\beta^*, u, v) = \phi_{ij} - \phi_{ji}$ can be rewritten as

$$G(\beta^*, u, v) = \frac{1 - \Pi_{k=1}^{N-1} \frac{g(-\beta^*(uk+v))}{g(+\beta^*(uk+v))}}{1 + \sum_{i=1}^{N-1} \Pi_{k=1}^{i} \frac{g(-\beta^*(uk+v))}{g(+\beta^*(uk+v))}}. \tag{8}$$

For $u = 0$, we have

$$G(\beta^*, 0, v) = \frac{1 - \Pi_{k=1}^{N-1} \frac{g(-\beta^* v)}{g(+\beta^* v)}}{1 + \sum_{i=1}^{N-1} \Pi_{k=1}^{i} \frac{g(-\beta^* v)}{g(+\beta^* v)}}. \tag{9}$$

Let $x = \frac{g(-\beta^* v)}{g(+\beta^* v)}$, which is always positive. We rewrite $G(\beta^*, 0, v) = c^*$ by $f(x) = 0$, where

$$f(x) = (c^* + 1)x^{N-1} + \sum_{i=1}^{N-2} c^* x^i + (c^* - 1). \tag{10}$$

Taking into account that $-1 \leq c^* \leq 1$, there is exactly once of the change of the signs between two consecutive coefficients of the polynomial $f(x)$. By the Descartes' Rule of Signs, there is exactly one positive root of Eq. (10), x^*. Besides, since $g(x)$ is strictly increasing and nonnegative, $g(-x)/g(x)$ is a strictly decreasing function. In addition, since $g(x) \to 1$ and $g(-x) \to 0$ as $x \to \infty$, we have $g(-x)/g(x) \to 0$, as $x \to +\infty$ while $g(-x)/g(x) \to +\infty$, as $x \to -\infty$. Therefore $g(-x)/g(x)$ is a bijection from $(-\infty, +\infty)$ to $(0, +\infty)$. In particular, for $x^* > 0$, there exists a unique v_0 such that $g(-\beta^* v_0)/g(\beta^* v_0) = x^*$. In other words $G(\beta^*, 0, v_0) = c^*$.

Second, we prove the existence of the implicit function. Denote $F(u, v) = G(\beta^*, u, v) - c^*$. Since $g(x)$ is continuously differentiable by assumption, $F(u, v)$ is continuously differentiable in the whole $u - v$ plane for any $-1 < c^* < 1$. we only need to show that $\frac{\partial F}{\partial v}|_{(0,v_0)} = \frac{\partial G}{\partial v}|_{(0,v_0)} \neq 0$.

Let $a_i(u, v) = \sum_{k=1}^{i} \ln(\frac{g(-\beta^*(uk+v))}{g(+\beta^*(uk+v))})$. Then $\frac{\partial F}{\partial v}$ is given by

$$\frac{-\exp(a_{N-1})\frac{\partial a_{N-1}}{\partial v}\left(1 + \sum_{i=1}^{N-1} \exp(a_i)\right) - (1 - \exp(a_{N-1}))\left(\sum_{i=1}^{N-1} \frac{\partial a_i}{\partial v} \exp(a_i)\right)}{\left(1 + \sum_{i=1}^{N-1} \exp\left(a_i\right)\right)^2}. \tag{11}$$

The sign of $\frac{\partial F}{\partial v}|_{(0,v_0)}$ is determined by the numerator of Eq. (11).

Classifying c^* by its signs, we will prove the existence of the implicit function $v = v(u)$.

For $c^* = 0$, then $G(\beta^*, 0, 0) = 0$, thus $v = 0$ is a solution of $G(\beta^*, 0, v) = 0$. Considering that the uniqueness of the solution which has been proved above, we have $v_0 = 0$. In this case, we have $1 - \exp(a_{N-1}) = 0$. By Eq. (11), the sign of the numerator of Eq. (11) is determined by $-\frac{\partial a_{N-1}}{\partial v}|_{(0,0)} =$

$2\beta^*(N-1)g'(0)/g(0)$, which is not zero. By the implicit function theorem, there exists a function $v(u)$ defined in a vicinity of $u=0$, such that $G(\beta^*, u, v(u)) = c^*$.

For $c^* \in (0,1)$, $G(\beta^*, 0, v_0) = c^*$ can be rewritten as $1 + \sum_{i=1}^{N-1} \exp(a_i)\Big|_{(0,v_0)} = (1-\exp(a_{N-1}))|_{(0,v_0)}/c^*$. We take these into the numerator of Eq. (11), then the numerator becomes

$$\frac{1-\exp(a_{N-1})}{-c^*}\left((1+c^*)\exp(a_{N-1})\frac{\partial a_{N-1}}{\partial v} + c^*\sum_{i=1}^{N-2}\exp(a_i)\frac{\partial a_i}{\partial v}\right)\Bigg|_{(0,v_0)}. \tag{12}$$

Since $\frac{1-\exp(a_{N-1})}{-c^*} = -(1+\sum_{i=1}^{N-1}\exp(a_i))$ is always negative, the sign of the numerator is opposite to that of the term in the bracket of Eq. (12). Remembering $a_i(u,v) = \sum_{k=1}^{i}\ln(\frac{g(-\beta^*(uk+v))}{g(+\beta^*(uk+v))})$, we have $\frac{\partial a_i}{\partial v}|_{(0,v_0)} = -\beta^* i\left[\frac{g'(-\beta^* v_0)}{g(-\beta^* v_0)} + \frac{g'(\beta^* v_0)}{g(\beta^* v_0)}\right]$. Let $y = g(-\beta^* v_0)/g(\beta^* v_0)$, the term in the bracket of Eq. (12) is

$$-\beta^*\underbrace{\left[\frac{g'(-\beta^* v_0)}{g(-\beta^* v_0)} + \frac{g'(\beta^* v_0)}{g(\beta^* v_0)}\right]}_{D_1}\underbrace{\left((1+c^*)(N-1)y^{N-1} + \sum_{k=1}^{N-2}c^* k y^k\right)}_{D_2}. \tag{13}$$

Considering that the imitation function $g(x)$ is always increasing and positive, D_1 is positive. Since $c^* > 0$ and $y > 0$ due to the fact that $y = g(-\beta^* v_0)/g(\beta^* v_0)$, D_2 is always positive. Therefore for $c^* \in (0,1)$, we have that $F(0, v_0) = 0$ and $\frac{\partial F}{\partial v}|_{(0,v_0)} \neq 0$. By employing the implicit function theorem, there exists $\delta > 0$ and a function $v(u)$ defined in $(-\delta, \delta)$, such that $G(\beta^*, u, v(u)) = c^*$.

For $c^* \in (-1, 0)$, instead of considering $G(\beta^*, u, v) = \phi_{ij} - \phi_{ji} = c^*$, we alter the name of strategy i and j, then we have that $G(\beta^*, \tilde{u}, \tilde{v}) = \phi_{ji} - \phi_{ij}$, where $\tilde{u} = u$ and $v + \tilde{v} = -Nu$. For $-c^* > 0$, and $\beta^* > 0$, by the above proof, there exists a function $\tilde{v} = \tilde{v}(\tilde{u})$ defined in a vicinity of $\tilde{u} = 0$ such that $G(\beta^*, \tilde{u}, \tilde{v}(\tilde{u})) = -c^*$. Taking into account that $\tilde{u} = u$ and $v + \tilde{v} = -Nu$, let $v(u) = -Nu - \tilde{v}(u)$, we have that the function $v(u)$ defined in the vicinity of $u = 0$ fulfills $G(\beta^*, u, v(u)) = c^*$.

In summary, we have proved that for arbitrary $c^* \in (-1,1)$ and $\beta^* > 0$, there exists a function defined in a vicinity of $u = 0$ such that $G(\beta^*, u, v(u)) = c^*$. Also by the implicit function theorem, $v(u)$ is continuous. Furthermore, since $g(x)$ is continuously differentiable, $\frac{\partial F}{\partial u}$ is continuous in the whole plane. This leads to that $v(u)$ is also differentiable. In fact, since $g(x)$ is differentiable, $v'(u)$ is continuous.

Finally we prove that if $0 < c^* < 1$, $v(0) > 0$.

In fact, by Eq. (10), we have $f(0) = c^* - 1 < 0$ and $f(1) = Nc^* > 0$. Since $f(x)$ is continuous in $[0,1]$, there is a $\tilde{x} \in (0,1)$ such that $f(\tilde{x}) = 0$. As we have proved that there is only one positive root for $f(x) = 0$, $x^* = \tilde{x}$. Since $x^* = g(-\beta v_0)/g(+\beta v_0)$ and $0 < x^* < 1$, we have $v(0) = v_0 > 0$.

This completes the proof.

5.2 Lemma 2

Proof To prove the existence of the inverse function of $H(u,v)$ around $(0, v_0)$, where $v_0 > 0$, we are employing the inverse function theorem. What we need to prove is: (i) $H(u,v)$ is continuously differentiable at $(0, v_0)$; (ii) if $v_0 > 0$, the Jacobian matrix of $H(u,v)$ at $(0, v_0)$ is non-degenerate.

First, since $g(x)$ is second order continuously differentiable, $H(u,v)$ is continuously differentiable at $(0, v_0)$.

Second, we show that if $v_0 > 0$, the Jacobian matrix of $H(u,v)$ at $(0, v_0)$ is non-degenerate. We rewrite the function $G(\beta, u, v)$ as $\tilde{G}(\beta u, \beta v)$, i.e.,

$$\tilde{G}(x,y) = \frac{F_1(x,y)}{F_2(x,y)}, \tag{14}$$

where

$$F_1(x,y) = 1 - \exp\left[\sum_{k=1}^{N-1} \ln\left(\frac{g(-xk-y)}{g(xk+y)}\right)\right],$$
$$F_2(x,y) = 1 + \sum_{i=1}^{N-1} \exp\left[\sum_{k=1}^{i} \ln\left(\frac{g(-xk-y)}{g(xk+y)}\right)\right]. \tag{15}$$

The Jacobian matrix of $H(u,v)$ at $(0,v_0)$ is equivalent to that of $\tilde{G}(x,y)$ at $(0,\beta v_0)$, and it is given by

$$\begin{pmatrix} \tilde{G}^{(1,0)}(0,\beta v_0) + \beta v_0 \tilde{G}^{(1,1)}(0,\beta v_0) & \tilde{G}^{(0,1)}(0,\beta v_0) + \beta v_0 \tilde{G}^{(0,2)}(0,\beta v_0) \\ \beta \tilde{G}^{(1,0)}(0,\beta v_0) & \beta \tilde{G}^{(0,1)}(0,\beta v_0) \end{pmatrix}, \tag{16}$$

where $\tilde{G}^{(i,j)} = \frac{\partial^{i+j}\tilde{G}}{\partial x^i \partial y^j}$.

In order to prove that Eq. (16) is non-degenerate, we perform elementary transformations, which do not change the rank of matrix.

Since β is positive, we multiply the second row of Eq. (16) with factor $-1/\beta$, and add it to the first row. This leads to

$$\begin{pmatrix} \beta v_0 \tilde{G}^{(1,1)}(0,\beta v_0) & \beta v_0 \tilde{G}^{(0,2)}(0,\beta v_0) \\ \beta \tilde{G}^{(1,0)}(0,\beta v_0) & \beta \tilde{G}^{(0,1)}(0,\beta v_0) \end{pmatrix}. \tag{17}$$

For Eq. (17), since $v_0 > 0$ by assumption, we multiply $1/(\beta v_0)$ to the first row then $1/\beta$ to the second row. This yields

$$\begin{pmatrix} \tilde{G}^{(1,1)}(0,\beta v_0) & \tilde{G}^{(0,2)}(0,\beta v_0) \\ \tilde{G}^{(1,0)}(0,\beta v_0) & \tilde{G}^{(0,1)}(0,\beta v_0) \end{pmatrix}. \tag{18}$$

Taking into account Eqs. (14) and (15), Eq. (18) is

$$\begin{pmatrix} \frac{S_{11}}{F_2^3} & \frac{S_{12}}{F_2^3} \\ \frac{S_{21}}{F_2^2} & \frac{S_{22}}{F_2^2} \end{pmatrix}, \tag{19}$$

where

$$\begin{aligned} S_{11} &= {F_1}^{(1,1)}(0,\beta v_0)F_2(0,\beta v_0)^2 - {F_2}^{(0,1)}(0,\beta v_0){F_1}^{(1,0)}(0,\beta v_0)F_2(0,\beta v_0) \\ &\quad - {F_1}^{(0,1)}(0,\beta v_0){F_2}^{(1,0)}(0,\beta v_0)F_2(0,\beta v_0) - F_1(0,\beta v_0){F_2}^{(1,1)}(0,\beta v_0)F_2(0,\beta v_0) \\ &\quad + 2F_1(0,\beta v_0){F_2}^{(0,1)}(0,\beta v_0){F_2}^{(1,0)}(0,\beta v_0) \\ S_{12} &= {F_1}^{(0,2)}(0,\beta v_0)F_2(0,\beta v_0)^2 - 2{F_1}^{(0,1)}(0,\beta v_0){F_2}^{(0,1)}(0,\beta v_0)F_2(0,\beta v_0) \\ &\quad - F_1(0,\beta v_0){F_2}^{(0,2)}(0,\beta v_0)F_2(0,\beta v_0) + 2F_1(0,\beta v_0){F_2}^{(0,1)}(0,\beta v_0)^2 \\ S_{21} &= F_2(0,\beta v_0){F_1}^{(1,0)}(0,\beta v_0) - F_1(0,\beta v_0){F_2}^{(1,0)}(0,\beta v_0) \\ S_{22} &= F_2(0,\beta v_0){F_1}^{(0,1)}(0,\beta v_0) - F_1(0,\beta v_0){F_2}^{(0,1)}(0,\beta v_0). \end{aligned} \tag{20}$$

By Eq. (15), F_2 is always positive thus non-zero. Multiplying F_2^3 to the first row of Eq. (19) then F_2^2 to the second row of Eq. (19) do not alter the rank of Eq. (19), and it leads to

$$J = \begin{pmatrix} S_{11} & S_{12} \\ S_{21} & S_{22} \end{pmatrix}. \tag{21}$$

Taking Eqs. (15) into consideration leads to

$$
\begin{aligned}
F_1(0,\beta v_0) =& 1 - h^{N-1} \\
{F_1}^{(1,0)}(0,\beta v_0) =& \left(h^{N-1}-1\right) a \sum_{i=1}^{N-1} i \\
{F_1}^{(0,1)}(0,\beta v_0) =& a(N-1)\left(h^{N-1}-1\right) \\
{F_1}^{(1,1)}(0,\beta v_0) =& (h^{N-1}-1)a^2(N-1)\sum_{i=1}^{N-1} i + (h^{N-1}-1)b\sum_{i=1}^{N-1} i \\
{F_1}^{(0,2)}(0,\beta v_0) =& (h^{N-1}-1)\left(a\sum_{i=1}^{N-1} i\right)^2 + (h^{N-1}-1)b(N-1) \\
F_2(0,\beta v_0) =& 1 + \sum_{i=1}^{N-1} h^i \\
{F_2}^{(1,0)}(0,\beta v_0) =& a\sum_{k=1}^{N-1} h^k \left(\sum_{i=1}^{k} i\right) \\
{F_2}^{(0,1)}(0,\beta v_0) =& a\sum_{k=1}^{N-1} kh^k \\
{F_2}^{(1,1)}(0,\beta v_0) =& \sum_{k=1}^{N-1}\left(a^2h^k k\sum_{i=1}^{k} i + bh^k\sum_{i=1}^{k} i\right) \\
{F_2}^{(0,2)}(0,\beta v_0) =& \sum_{k=1}^{N-1}\left(a^2k^2h^k + bkh^k\right),
\end{aligned}
\tag{22}
$$

where

$$
\begin{aligned}
h =& \frac{g(-\beta v_0)}{g(\beta v_0)} \\
a =& -\frac{g'(-\beta v_0)}{g(-\beta v_0)} - \frac{g'(\beta v_0)}{g(\beta v_0)} \\
b =& \frac{g''(-\beta v_0)g(-\beta v_0) - (g'(-\beta v_0))^2}{(g(-\beta v_0))^2} - \frac{g''(\beta v_0)g(\beta v_0) - (g'(\beta v_0))^2}{(g(\beta v_0))^2}.
\end{aligned}
\tag{23}
$$

Considering the following identities [11]

$$
\begin{aligned}
\sum_{i=1}^{k} i &= \frac{k(k+1)}{2} \\
\sum_{i=1}^{k} i^2 &= \frac{k(k+1)(2k+1)}{6} \\
\sum_{i=1}^{k} h^i &= \frac{h-h^{k+1}}{1-h} = f_k(h) \\
\sum_{i=1}^{k} ih^i &= hf_k'(h) \\
&= \frac{((h-1)N-1)h^{N+1}+h}{(h-1)^2} \\
\sum_{i=1}^{k} i^2h^i &= h\frac{d}{dh}\left(hf_k'(h)\right) \\
&= \frac{h\left(\left(-2N^2-2N+1\right)h^{N+1}+N^2h^{N+2}+(N+1)^2h^N-h-1\right)}{(h-1)^3} \\
\sum_{i=1}^{k} i^3h^i &= h\frac{d}{dh}\left[h\frac{d}{dh}\left(hf_k'(h)\right)\right] \\
&= (h-1)^{-4}h(N^3h^{N+3}+(3N^3+6N^2-4)h^{N+1} \\
&- (3N^3+3N^2-3N+1)h^{N+2}-(N+1)^3h^N+h^2+4h+1),
\end{aligned}
\tag{24}
$$

and taking them into Eqs. (22) lead to

$$
\begin{aligned}
F_1(0,\beta v_0) =& 1-h^{N-1}\\
{F_1}^{(1,0)}(0,\beta v_0) =& \frac{1}{2}a(N-1)N\left(h^{N-1}-1\right)\\
{F_1}^{(0,1)}(0,\beta v_0) =& a(N-1)\left(h^{N-1}-1\right)\\
{F_1}^{(1,1)}(0,\beta v_0) =& \frac{1}{2}a^2N(N-1)^2\left(h^{N-1}-1\right)+\frac{1}{2}bN(N-1)\left(h^{N-1}-1\right)\\
{F_1}^{(0,2)}(0,\beta v_0) =& \frac{1}{4}a^2(N-1)^2N^2\left(h^{N-1}-1\right)+b(N-1)\left(h^{N-1}-1\right)\\
F_2(0,\beta v_0) =& \frac{h^N-h}{h-1}+1\\
{F_2}^{(1,0)}(0,\beta v_0) =& \frac{a\left(-2N^2h^{N+1}+N^2h^{N+2}+2h^{N+1}-Nh^{N+2}+N^2h^N+Nh^N-2h\right)}{2(h-1)^3}\\
{F_2}^{(0,1)}(0,\beta v_0) =& \frac{a\left(Nh^{N+1}-h^{N+1}-Nh^N+h\right)}{(h-1)^2}\\
{F_2}^{(1,1)}(0,\beta v_0) =& (-2a^2h^{N+1}-4a^2h^{N+2}+4a^2h^2+2a^2h-2bh^{N+1}+2bh^{N+2}-2bh^2+2bh\\
&+N\left(-5a^2h^{N+1}+4a^2h^{N+2}+a^2h^{N+3}+bh^{N+1}+bh^{N+2}-bh^{N+3}-bh^N\right)\\
&+N^2\left(3a^2h^{N+2}-2a^2h^{N+3}-a^2h^N+3bh^{N+1}-3bh^{N+2}+bh^{N+3}-bh^N\right)\\
&+N^3\left(3a^2h^{N+1}-3a^2h^{N+2}+a^2h^{N+3}-a^2h^N\right))[(2^{-1}(h-1)^{-4})]\\
{F_2}^{(0,2)}(0,\beta v_0) =& (a^2h^{N+1}+a^2h^{N+2}-a^2h^2-a^2h+bh^{N+1}-bh^{N+2}+bh^2-bh\\
&+N\left(2a^2h^{N+1}-2a^2h^{N+2}-2bh^{N+1}+bh^{N+2}+bh^N\right)\\
&+N^2\left(-2a^2h^{N+1}+a^2h^{N+2}+a^2h^N\right))(h-1)^{-3}. \qquad (25)
\end{aligned}
$$

On one hand, $g(x)$ is increasing and positive, $v_0>0$ thus $0<h<1$, where $h=g(-v_0\beta)/g(-v_0\beta)$ in Eq. (23). On the other hand, a, b and h in Eq. (23) are not dependent on population size N, they are viewed as order 1. Therefore, for any $i>0$, $N^ih^N\to 0$ as the large population size N is sufficiently

large. Since the population size N is large by assumption, Eqs. (25) can be approximated by

$$
\begin{aligned}
F_1(0,\beta v_0) \approx& 1\\
{F_1}^{(1,0)}(0,\beta v_0) \approx& -\frac{1}{2}a(N-1)N\\
{F_1}^{(0,1)}(0,\beta v_0) \approx& -a(N-1)\\
{F_1}^{(1,1)}(0,\beta v_0) \approx& -\frac{1}{2}a^2N(N-1)^2-\frac{1}{2}bN(N-1)\\
{F_1}^{(0,2)}(0,\beta v_0) \approx& -\frac{1}{4}a^2(N-1)^2N^2-b(N-1)\\
F_2(0,\beta v_0) \approx& \frac{-1}{h-1}\\
{F_2}^{(1,0)}(0,\beta v_0) \approx& \frac{-ah}{(h-1)^3}\\
{F_2}^{(0,1)}(0,\beta v_0) \approx& \frac{ah}{(h-1)^2}\\
{F_2}^{(1,1)}(0,\beta v_0) \approx& \frac{h^2(2a^2-b)+h(a^2+b)}{(h-1)^4}\\
{F_2}^{(0,2)}(0,\beta v_0) \approx& \frac{b(h-1)h-a^2h(h+1)}{(h-1)^3}.
\end{aligned} \tag{26}
$$

Taking these approximations into Eq. (20), we find that the determinant of Eq. (21) is of order N^6 for large population size, i.e.,

$$
|J| \sim \frac{a^3}{8(1-h)^3}N^6. \tag{27}
$$

For large population size the sign of the determinant of J is determined by $a^3/(8(1-h)^3)$. Since $v_0>0$ by assumption, by Eq. (23) we have that $0<h<1$ and $a<0$. Thus $a^3/(8(1-h)^3)$ is negative and non-zero, which leads to the non-zero determinant of J. Regarding J is derived by a series of elementary transformations of the Jacobian matrix of $H(u,v)$ at $(0,v_0)$, the Jacobian matrix of $H(u,v)$ at $(0,v_0)$ is also non-degenerate. By the inverse function theorem, there is a $\delta>0$ such that $H(u,v)$ is invertible in the vicinity of $(0,v_0)$, $B((0,v_0))$, where $B((0,v),\delta)=\{(u,v)|\sqrt{u^2+(v-v_0)^2}<\delta\}$.

This completes the proof.

5.3 Lemma 3

Proof By the definition of $u_{i,i+1}$ and $v_{i,i+1}$ in Section 1, we have

$$
\begin{aligned}
u_{i,i+1} &= \frac{1}{N-1}a_{ii}-\frac{1}{N-1}a_{i,i+1}-\frac{1}{N-1}a_{i+1,i}+\frac{1}{N-1}a_{i+1,i+1},\\
v_{i,i+1} &= -\frac{1}{N-1}a_{ii}+\frac{N}{N-1}a_{i,i+1}-a_{i+1,i+1},
\end{aligned} \tag{28}
$$

where $i=1,2,3$.

Eq. (28) can be rewritten as

$$
Pl=q, \tag{29}
$$

where

$$P = \frac{1}{N-1} \underbrace{\begin{pmatrix} 1 & -1 & 0 & -1 & 1 & 0 & 0 & 0 & 0 \\ -1 & N & 0 & 0 & -N+1 & 0 & 0 & 0 & 0 \\ 0 & 0 & 0 & 0 & 1 & -1 & 0 & -1 & 1 \\ 0 & 0 & 0 & 0 & -1 & N & 0 & 0 & -N+1 \\ 1 & 0 & -1 & 0 & 0 & 0 & -1 & 0 & 1 \\ -N+1 & 0 & 0 & 0 & 0 & 0 & N & 0 & -1 \end{pmatrix}}_{Q}, \tag{30}$$

$$l = (a_{11}, a_{12}, a_{13}, a_{21}, a_{22}, a_{23}, a_{31}, a_{32}, a_{33})^T, \tag{31}$$

and

$$q = (u_{12}, v_{12}, u_{23}, v_{23}, u_{31}, v_{31})^T. \tag{32}$$

By assumption $N > 1$, thus $r(P) = r(Q)$, i.e., the rank of P is the same as that of Q. On the one hand, $Q \in R^{6\times 9}$, the rank of Q is no more than 6, i.e., $r(Q) \leq 6$. On the other hand, we eliminate the first, the fifth and the ninth columns of Q, and arrive at a sub matrix of Q, i.e. a 6×6 matrix,

$$Q_1 = \begin{pmatrix} -1 & 0 & -1 & 0 & 0 & 0 \\ N & 0 & 0 & 0 & 0 & 0 \\ 0 & 0 & 0 & -1 & 0 & -1 \\ 0 & 0 & 0 & N & 0 & 0 \\ 0 & -1 & 0 & 0 & -1 & 0 \\ 0 & 0 & 0 & 0 & N & 0 \end{pmatrix}. \tag{33}$$

Note the determinant of Q_1 is $-N^3$ which is nonzero, the rank of Q_1 is six, i.e., $r(Q_1) = 6$. Considering that Q_1 is obtained via eliminating the columns of Q, $r(Q_1) \leq r(Q)$. In summary, $6 = r(Q_1) \leq r(Q) \leq 6$. This leads to $r(Q) = r(P) = 6$. By elementary linear algebra, we have that all the solutions of $Pl = 0$ forms a linear space, i.e., Ker$P$, of dimension $\mathrm{Dim}(l) - r(P) = 3$, where $\mathrm{Dim}(l)$ is the dimension of vector l and $r(P)$ is the rank of matrix P. Furthermore, by assumption $u_{i,i+1}$ and $v_{i,i+1}$ are not all zero, q in Eq. (32) as a vector is non-zero, all the solutions of $Pl = q$ are presented as

$$\{l^* + sol | sol \in \mathrm{Ker}P\}, \tag{34}$$

where l^* is a special solution of $Pl = q$.

In other words, for a given $N > 1$, not-all-zero $u_{i,i+1}$ and $v_{i,i+1}$, all the solutions of $Pl = q$ forms an affine space of dimension 3. Regarding that $l = (a_{11}, a_{12}, a_{13}, a_{21}, a_{22}, a_{23}, a_{31}, a_{32}, a_{33})^T$, it corresponds to a 3×3 matrix.

This completes the proof.

5.4 Lemma 4

Proof For $n = 3$, the uniform distribution is the stationary distribution of M at β^*, if and only if

$$\frac{1}{3} \begin{pmatrix} 1 \\ 1 \\ 1 \end{pmatrix}^T (M - I_3) \Bigg|_{\beta^*} = 0. \tag{35}$$

Considering that

$$(M-I_3)|_{\beta^*}=\frac{\mu}{3}\left(\begin{array}{ccc}-\phi_{12}-\phi_{13} & \phi_{12} & \phi_{13}\\ \phi_{21} & -\phi_{21}-\phi_{23} & \phi_{23}\\ \phi_{31} & \phi_{32} & -\phi_{31}-\phi_{32}\end{array}\right)\Bigg|_{\beta^*}, \tag{36}$$

with non-vanishing mutation rate μ, Eq. (35) is equivalent to

$$\left(\begin{array}{c}1\\1\\1\end{array}\right)^T\left(\begin{array}{ccc}-\phi_{12}-\phi_{13} & \phi_{12} & \phi_{13}\\ \phi_{21} & -\phi_{21}-\phi_{23} & \phi_{23}\\ \phi_{31} & \phi_{32} & -\phi_{31}-\phi_{32}\end{array}\right)\Bigg|_{\beta^*}=0, \tag{37}$$

or

$$\left(\begin{array}{c}(\phi_{31}-\phi_{13})-(\phi_{12}-\phi_{21})\\ (\phi_{12}-\phi_{21})-(\phi_{23}-\phi_{32})\\ (\phi_{23}-\phi_{32})-(\phi_{31}-\phi_{13})\end{array}\right)\Bigg|_{\beta^*}=0. \tag{38}$$

Eq. (38) is equivalent to

$$\phi_{12}-\phi_{21}=\phi_{23}-\phi_{32}=\phi_{31}-\phi_{13}, \tag{39}$$

i.e., at β^*

$$G_{12}=G_{23}=G_{31}. \tag{40}$$

Notice that every step of the above proof is either "if and only if" or "be equivalent with", Eq. (40) is the necessary and sufficient condition of that the stationary distribution of M is uniform at β^*.

This completes the proof.

5.5 Lemma 5

Proof Making use of $x=\exp(\ln(x))$ as well as $\Pi\exp(x)=\exp(\sum x)$, for 2×2 games, we rewrite the fixation probability ϕ_{12} in Eq. (4) as

$$\phi_{12}=\frac{1}{1+\sum_{i=1}^{N-1}\exp\left[\sum_{k=1}^{i}\ln(g(-\beta(u_{12}k+v_{12})))-g(+\beta(u_{12}k+v_{12})))\right]}. \tag{41}$$

Since $u_{12}>0$ and $v_{12}>0$ by assumption, $u_{12}k+v_{12}>0$ for $1\le k\le N-1$. Taking into account that $g(x)\to 1$ and $g(-x)\to 0$ as $x\to+\infty$, we have that for $1\le k\le N-1$, $\ln(g(-\beta(u_{12}k+v_{12})))-g(+\beta(u_{12}k+v_{12})))\to-\infty$ as $\beta\to+\infty$. Taking this into Eq. (41) leads to that $\phi_{12}\to 1$ as $\beta\to+\infty$.

By the same argument, we have $\sum_{k=1}^{N-1}\ln(g(-\beta(u_{12}k+v_{12})))-g(+\beta(u_{12}k+v_{12})))\to-\infty$ as $\beta\to+\infty$. By Eq. (6), we have $\phi_{21}/\phi_{12}\to 0$ as $\beta\to+\infty$. Therefore $\phi_{21}=(\phi_{21}/\phi_{12})\phi_{12}\to 0$ as $\beta\to+\infty$.

This completes the proof.

5.6 Lemma 6

Proof By assumption (i), there is an $i\in\{1,2,3\}$ such that $\left(\frac{\partial}{\partial\beta}G_{i,i+1}-\frac{\partial}{\partial\beta}G_{i+1,i+2}\right)\Big|_{\beta^*}\neq 0$, the vector h is non-zero, where

$$h=\left(\begin{array}{c}\frac{\partial}{\partial\beta}G_{1,2}-\frac{\partial}{\partial\beta}G_{2,3}\\ \frac{\partial}{\partial\beta}G_{2,3}-\frac{\partial}{\partial\beta}G_{3,1}\\ \frac{\partial}{\partial\beta}G_{3,1}-\frac{\partial}{\partial\beta}G_{1,2}\end{array}\right)^T\Bigg|_{\beta^*}. \tag{42}$$

Considering that $G_{ij} = \phi_{ij} - \phi_{ji}$, the vector h can be rewritten as

$$\left(\begin{array}{c}1\\1\\1\end{array}\right)^T \frac{\partial}{\partial\beta}\left(\begin{array}{ccc}-\phi_{12}-\phi_{13} & \phi_{12} & \phi_{13}\\ \phi_{21} & -\phi_{21}-\phi_{23} & \phi_{23}\\ \phi_{31} & \phi_{32} & -\phi_{31}-\phi_{32}\end{array}\right)\Bigg|_{\beta^*} \tag{43}$$

or

$$h = \frac{3}{\mu}\left(\begin{array}{c}1\\1\\1\end{array}\right)^T \left(\frac{\partial}{\partial\beta}(M - I_3)\right)\bigg|_{\beta^*}, \tag{44}$$

where M is the transition matrix, i.e., Eq. (2) for $n = 3$, μ is the non-vanishing mutation rate and I_3 is the identity matrix of order 3.

By assumption ii), $z = (z_1, z_2, z_3) \in R^{1\times 3}$ is the stationary distribution of M, z is a function of β and $z(M - I_3) = 0$ for all $\beta > 0$. Since $g(x)$ is differentiable by assumption, we take the derivative of the function $z(M - I_3)$ at β^*. This leads to

$$\left(\left(\frac{\partial}{\partial\beta}z\right)(M - I_3) + z\left(\frac{\partial}{\partial\beta}(M - I_3)\right)\right)\bigg|_{\beta^*} = 0. \tag{45}$$

Regarding M bears the uniform distribution as the stationary distribution at β^* by assumption, i.e., $z = 1/3(1, 1, 1)$ at β^*, Eq. (45) can be rewritten as

$$\left(\frac{\partial}{\partial\beta}z\right)(M - I_3)\bigg|_{\beta^*} = -\frac{\mu}{9}h. \tag{46}$$

Since h is non-zero at β^*, $\frac{\partial}{\partial\beta}z$ is nonzero at β^*.

Furthermore, we prove that there exists j^* such that $\frac{\partial}{\partial\beta}z_{j^*} \neq \frac{\partial}{\partial\beta}z_{j^*+1}$ at β^*. Otherwise $\frac{\partial}{\partial\beta}z_k = \frac{\partial}{\partial\beta}z_{k+1}$ at β^* for $k = 1, 2, 3$. Considering that $\sum_{k=1}^3 z_k = 1$ for all β, $\sum_{k=1}^3 \frac{\partial}{\partial\beta}z_k = 0$ at β^*. This leads to $\frac{\partial}{\partial\beta}z_k = 0$ for $k = 1, 2, 3$ at β^*, i.e., $\frac{\partial}{\partial\beta}z$ is a zero vector at β^*. This is a contradiction.

For this j^*, we have $\frac{\partial}{\partial\beta}(z_{j^*} - z_{j^*+1}) \neq 0$ at β^*, without loss of generality, we assume $\frac{\partial}{\partial\beta}(z_{j^*} - z_{j^*+1}) > 0$ at β^*. Since the imitation function is continuously differentiable, $\frac{\partial}{\partial\beta}(z_{j^*} - z_{j^*+1})$ is continuous at β^*. This implies that there exists $\delta > 0$ such that $\frac{\partial}{\partial\beta}(z_{j^*} - z_{j^*+1}) > 0$ for all $\beta \in (\beta^* - \delta, \beta^* + \delta)$. Thus $z_{j^*} - z_{j^*+1}$ is increasing within the interval $(\beta^* - \delta, \beta^* + \delta)$. Considering that the uniform distribution is the stationary distribution at $\beta = \beta^*$, $z_{j^*} - z_{j^*+1} = 0$ at β^* by assumption, we have $z_{j^*} - z_{j^*+1} < 0$ for $\beta \in (\beta^* - \delta, \beta)$ and $z_{j^*} - z_{j^*+1} > 0$ for $\beta \in (\beta, \beta^* + \delta)$. In other words, the ranking of strategy j^* and $j^* + 1$ changes at β^*.

This completes the proof.

References

1. Fudenberg D, Imhof LA (2006) Imitation processes with small mutations. Journal of Economic Theory 131: 251–262.

2. Wu B, Gokhale CS, Wang L, Traulsen A (2012) How small are small mutation rates? Journal of Mathematical Biology 64: 803-827.

3. García J, Traulsen A (2012) The structure of mutations and the evolution of cooperation. PLoS One 7: e35287.

4. Gardiner CW (2004) Handbook of Stochastic Methods. Springer, NY, third edition.

5. Ewens WJ (2004) Mathematical Population Genetics. I. Theoretical Introduction. New York: Springer.

6. Wu B, Altrock PM, Wang L, Traulsen A (2010) Universality of weak selection. Physical Review E 82: 046106.

7. Antal T, Nowak MA, Traulsen A (2009) Strategy abundance in 2×2 games for arbitrary mutation rates. Journal of Theoretical Biology 257: 340–344.

8. Nowak MA (2006) Evolutionary Dynamics. Cambridge MA: Harvard University Press.

9. Karlin S, Taylor HMA (1975) A First Course in Stochastic Processes. London: Academic, 2nd edition edition.

10. Traulsen A, Hauert C (2009) Stochastic evolutionary game dynamics. In: Schuster HG, editor, Reviews of Nonlinear Dynamics and Complexity, Weinheim: Wiley-VCH, volume II. pp. 25-61.

11. Graham RL, Knuth DE, Patashnik O (1994) Concrete Mathematics. Addison-Wesley, second edition.
